# Autophagy-mediated metabolic effects of aspirin

**DOI:** 10.1038/s41420-020-00365-0

**Published:** 2020-11-24

**Authors:** Francesca Castoldi, Juliette Humeau, Isabelle Martins, Sylvie Lachkar, Damarys Loew, Florent Dingli, Sylvère Durand, David Enot, Noëlie Bossut, Alexis Chery, Fanny Aprahamian, Yohann Demont, Paule Opolon, Nicolas Signolle, Allan Sauvat, Michaela Semeraro, Lucillia Bezu, Elisa Elena Baracco, Erika Vacchelli, Jonathan G. Pol, Sarah Lévesque, Norma Bloy, Valentina Sica, Maria Chiara Maiuri, Guido Kroemer, Federico Pietrocola

**Affiliations:** 1 Centre de Recherche des Cordeliers, INSERM U1138, Team “Meta7bolism, Cancer & Immunity”, Sorbonne Université, Université de Paris, Institut Universitaire de France, Paris, France; 2 Metabolomics and Cell Biology Platforms, Gustave Roussy Cancer Campus, Villejuif, France; 3 Institut Curie, PSL Research University, Centre de Recherche, Laboratoire de Spectrométrie de Masse Protéomique, 26 rue d’Ulm, Paris, 75248 Cedex 05 France; 4 Department of Experimental Pathology, INSERM Unit U981, Gustave Roussy, Université Paris-Sud Saclay, Villejuif, France; 5 Centre d’Investigation Clinique-Unite de Recherche Clinique Paris Centre Necker-Cochin, Assistance Publique-Hôpitaux de Paris, Paris, France; 6 Fondazione IRCCS Istituto Nazionale dei Tumori, Milan, Italy; 7 Department of Radiation Oncology, Weill Cornell Medical College, New York, NY USA; 8 Cell Biology Group, Department of Experimental and Health Sciences, Pompeu Fabra University (UPF), Barcelona, Spain; 9 Pôle de Biologie, Hôpital Européen Georges Pompidou, Assistance Publique-Hôpitaux de Paris, Paris, France; 10 Suzhou Institute for Systems Medicine, Chinese Academy of Medical Sciences, Suzhou, China; 11 Karolinska Institute, Department of Women’s and Children’s Health, Karolinska University Hospital, Stockholm, Sweden; 12 Karolinska Institute, Department of Bioscience and Nutrition, Huddinge, Sweden

**Keywords:** Metabolic disorders, Macroautophagy

## Abstract

Salicylate, the active derivative of aspirin (acetylsalicylate), recapitulates the mode of action of caloric restriction inasmuch as it stimulates autophagy through the inhibition of the acetyltransferase activity of EP300. Here, we directly compared the metabolic effects of aspirin medication with those elicited by 48 h fasting in mice, revealing convergent alterations in the plasma and the heart metabolome. Aspirin caused a transient reduction of general protein acetylation in blood leukocytes, accompanied by the induction of autophagy. However, these effects on global protein acetylation could not be attributed to the mere inhibition of EP300, as determined by epistatic experiments and exploration of the acetyl-proteome from salicylate-treated EP300-deficient cells. Aspirin reduced high-fat diet-induced obesity, diabetes, and hepatosteatosis. These aspirin effects were observed in autophagy-competent mice but not in two different models of genetic (*Atg4b*^−/−^ or *Bcln1*^+/−^) autophagy-deficiency. Aspirin also improved tumor control by immunogenic chemotherapeutics, and this effect was lost in T cell-deficient mice, as well as upon knockdown of an essential autophagy gene (*Atg5*) in cancer cells. Hence, the health-improving effects of aspirin depend on autophagy.

## Introduction

Aspirin is one of the oldest molecules to be used as a chemically defined entity for the treatment of human disease. Indeed, salicylate and its derivatives contained in plant extracts have been used since Mesopotamian times^1^. As true for many other pharmaceutical agents, the effects of aspirin have been determined empirically to include anti-inflammatory, analgesic, and thrombosis-preventive effects before a molecular mode of action would have been postulated^1^. Over the past decades, aspirin has been discovered to mediate prominent anti-arteriosclerotic effects, justifying its use for the prevention of myocardial infarction^2^. Moreover, aspirin reduces the frequency of many solid cancers, in particular gastrointestinal carcinomas^3–5^. Aspirin also attenuates the manifestations of metabolic syndrome in the context of obesity^6–8^. However, the many positive properties of aspirin are overshadowed by potentially life-threatening side effects, notably gastrointestinal bleeding, that preclude its population-wide, unrestricted use for the prevention of metabolic, inflammatory, cardiovascular, and neoplastic diseases^9^.

Aspirin (acetylsalicylate) and its active metabolite salicylate (generated by deacetylation of aspirin) have been shown to act on multiple distinct molecular targets. These include cyclooxygenases 1 and 2 (responsible for the generation of proinflammatory and platelet-aggregating prostaglandins and thromboxanes)^10^, but also a series of other proteins including the energy sensor AMP-activated protein kinase (AMPK)^11^ and the proinflammatory NF-kB (nuclear factor kappa-light-chain-enhancer of activated B cells) activating kinase IKKβ (inhibitor of nuclear factor kappa-B kinase subunit beta)^12,13^. Recently, we and others have shown that salicylate can also inhibit the acetyltransferase activity of E1A-associated protein p300 (EP300)^14–16^. By inhibiting EP300, aspirin stimulates autophagy, a cytoplasmic renewal process that has prominent cardioprotective and antineoplastic effects^17–20^, perhaps explaining the long-term effects of aspirin on the two major age-related disease categories, namely, cardiovascular disease and cancer.

Here, we investigated the metabolic effects of aspirin on the extracellular (plasma) and intracellular metabolome in mice by comparing them with those elicited by fasting, which is known to reduce protein acetylation and ignite autophagy in several tissues^20–22^. We show that aspirin induces metabolic changes that are commensurate with the alterations produced by nutrient starvation, that it can indeed cause protein deacetylation in circulating white blood cells, that it inhibits EP300 but also other protein acetyltransferases, and that it mediates its positive metabolic effects through the induction of autophagy. Indeed, the capacity of aspirin to reduce dietary adiposity, diabetes, and hepatosteatosis, as well as to enhance the efficacy of immunogenic chemotherapeutics, was lost in two distinct genetic mouse models of autophagy deficiency, as well as upon knockdown of *Atg5* autophagy gene in cancer cells, respectively.

## Experimental model and subject details

### Mouse strains and housing

Female and male wild-type C57BL/6, nude athymic (*nu/nu*) mice (obtained from Envigo, Gannat, France), *Atg4b*^−/−^ C57BL/6 mice (a gift of Pr. Carlos Lopez-Otin, University of Oviedo, Spain), and *Bcln1*^*−/+*^ C57BL/6 mice (a gift of Dr. Beth Levine, UT Southwestern Medical Center, Dallas, USA) were bred in Cordeliers Research Center. Animals were maintained according to the FELASA guidelines and local guidelines from the Animal Experimental Ethics Committee (#24938-2020040122381916v3; #10511-2017070511526660v2; #4462-2016031108383932v3; #03981.02; #2314-2015101617187579v1). Mice were housed in a specific pathogen-free condition and in a temperature-controlled environment with 12 h light/dark cycles and received chow diet (#R04; Safe, Augy, France) or high-fat diet (HFD; #260HF, Safe) and water ad libitum.

### Mouse experiments and tissue processing

For tumor growth experiments, 3 × 10^5^ wild-type (WT) MCA205, *Atg5*^KD^ MCA205, overexpressing CD39 MCA205 cells were inoculated subcutaneously (near the thigh) into WT or *nu/nu* C57BL/6 female mice, and tumor surface (longest dimension × perpendicular dimension) was routinely monitored using a common caliper. When the tumor surface reached 25–35 mm^2^, mice were treated either with 5.17 mg/kg i.p. mitoxantrone (MTX; M6545, Sigma Aldrich) in 100 μl in Phosphate Saline Buffer (PBS; #10010023, Thermo Fisher Scientific), 10 mg/kg i.p. oxaliplatin (OXA; O9512, Sigma Aldrich) in 100 μl PBS or an equivalent volume of PBS, alone or in combination with 100 mg/kg of acetylsalicylic acid (aspirin; #A5376, Sigma Aldrich), starting the day before the chemotherapy and then three times per week by i.p. administration in Earle’s Balanced Salts solution (EBSS; #E2888, Sigma Aldrich). For the weight gain experiment, starting from 7 weeks of age, male mice (from the reported genetic strain) were divided by simple randomization in two groups: (1) HFD (20% protein, 36% lipids, and 36.7% carbohydrate) and (2) HFD and gavage daily administration of 100 mg/kg of acetylsalicylic acid in EBSS solution. Mice were weighed once a week, to monitor their weight gain, and subjected to metabolic tests around the sixth/eleventh week of treatment. Then, based on the different settings, animals were sacrificed after 10–12 weeks of treatment by cervical dislocation and organs collected and processed for the histological analysis (see below). For the short-term autophagy induction studies in circulating leukocytes, mice were treated with aspirin (i.p., 100 mg/kg) for 6 h and, optionally, mice were injected with leupeptin (#Sp042217, Euromedex) i.p., 15 mg/kg 2 h before blood collection; after the indicated time, animals were anesthetized and 500 μL of blood were drawn by cardiac puncture, followed by euthanasia, and isolated leukocytes were prepared, as described below, for the immunoblotting or immunofluorescence staining. For the evaluation of aspirin-induced protein deacetylation, mice were treated with i.p. 100 mg/kg of aspirin and blood collected at different time point (0, 30, 60, 120, and 240 min post-administration), or treated with different dose of aspirin (i.p. injection of 10, 100, or 300 mg/kg) and blood collected after 1 h. In both settings, isolated leukocytes were prepared for the immunofluorescence staining. For the metabolome plasma analysis, mice were kept under standard conditions, or were left for 48 h in the absence of nutrients (with ad libitum access to drinking water, following standard procedures^23^) or treated for 6 h with aspirin (i.p. 100 mg/kg). After this period, animals were anesthetized and blood was drawn by cardiac puncture, followed by euthanasia. Experiments with mice were conducted with a minimum number of animals, in accordance with ARRIVE guidelines. Sample sizes were selected based on prior experience of the laboratory in metabolic studies. A minimum of three biological replicates was used for each experiment. Experiments were not conducted in blind conditions.

### Metabolomics analysis

#### Cultured cells preparation

Cells were cultured in 6-well plates being at an approximate 80% of confluence the day of the experiment. After the corresponding treatment, wells plates were placed upon ice under chemical hood and processed for extraction before injection, as described in ref. ^24^.

#### Sample preparation tissues

About 30 mg of tissues for each condition were first weighted and solubilized into 1.5 mL polypropylene microcentrifuge tubes, with 1 ml of cold lysate buffer (MeOH/Water/Chloroform, 9/1/1, −20 °C). They were then processed for GC-MS (gas chromatography-mass spectrometry) and LC-MS (liquid chromatography-mass spectrometry) injections as reported in^25^. Data associated with fasted animals are shared with ref. ^26^.

#### Sample preparation plasma (lithium heparin)

A volume of 50 µl of plasma for each condition was mixed with 500 ml of cold lysate buffer (MeOH/Water/Chloroform, 9/1/1, −20 °C). They were then processed for GCMS and LCMS injections as in^25^.

#### Targeted analysis of CoAs and nucleoside phosphates by ion pairing ultra-high performance liquid chromatography (UHPLC) coupled to a Triple Quadrupole (QQQ) mass spectrometer (Tissue and cultured cells)

Targeted analysis was performed on a RRLC 1260 system (Agilent Technologies, Waldbronn, Germany) coupled to a Triple Quadrupole 6410 (Agilent Technologies) equipped with an electrospray source operating in positive mode. The gas temperature was set to 350 °C with a gas flow of 12 L/min. The capillary voltage was set to 3.5 kV. The procedure was performed as in ref. ^24^.

#### Widely targeted analysis of intracellular metabolites gas chromatography (GC) coupled to a triple quadrupole (QQQ) mass spectrometer

The GC-MS/MS method was performed on a 7890B gas chromatography (Agilent Technologies, Waldbronn, Germany) coupled to a triple quadrupole 7000C (Agilent Technologies, Waldbronn, Germany) equipped with a High sensitivity electronic impact source (EI) operating in positive mode^24^.

#### Untargeted analysis of intracellular metabolites by ultra-high performance liquid chromatography (UHPLC) coupled to a quadrupole- time of flight (QTOF) mass spectrometer

Profiling of intracellular metabolites was performed on a Liquid Chromatography (LC) 1260 system (Agilent Technologies, Waldbronn, Germany) coupled to a QTOF 6520 (Agilent Technologies) equipped with an electrospray source operating in both positive and negative mode and full scan mode from 50 to 1000 Da^24^.

#### Quality control policy

A daily qualification of the instrumentation was set up with automatic tune and calibration processes. These qualifications were completed with double injections of standards mixes, at the beginning and at the end of the run, as for a blank extracted sample to control the background impurities. Mixtures were adapted for each chromatographic method. After the extraction, the pool of QC sample was used to passivize the column before the analysis with the proper biological matrix and re-injected during the batch to monitor and correct analytical bias occurring during the batch (*m/z*, retention time, and sensitivity drifts) during post-acquisition treatment signal.

#### Chemical products

Acetonitrile and Methanol are from HoneyWell. Isopropanol and Acetic acid were from VWR. Chloroform is from Arcos organics. Formic acid (FA), methoxyamine hydrochloride, *N*-Methyl-*N*-(trimethylsilyl) trifluoroacetamide (MSTFA), *N*-tert-butyldimethylsilyl-*N*-methyltrifluoroacetamide (MSTBFA), O-ethylhydroxylamine hydrochloride, pyridine, dibutylamine ammonium acetate (DBAA), sulfosalicylic acid (SSA), heptafluorobutyric acid (HFBA) are from Merck.

### Histological analysis

After treatment, mice were sacrificed by cervical dislocation followed by immediate fixation of the liver tissue and the visceral fat in 4% formaldehyde (#F8775, Sigma Aldrich) PBS solution. Fixed samples were embedded in paraffin, and 3 µm thick sections were stained with hematoxylin-eosin-saffranin (H&E). Each slide was examined using a Zeiss Axiophot microscope. Virtual Slide microscope VS120-SL (Olympus, Tokyo, Japan), 20X air objective (0.75 NA) was used to acquire histological slides. An in-house algorithm was developed with Image J software (National Institutes of Health, Bethesda, Maryland, USA), in order to quantify the hepatic steatosis damage and adipocytes size^27^. On rare occasions when technical issues with staining occurred, slides were excluded from histological analysis.

### Analysis of whole-body composition

Non-invasive determination of lean and fat tissue mass, and free fluid was performed on non-anesthetized mice using Time Domain-NMR technology (Minispec LF90II; Bruker BioSpin).

### Tolerance tests

Mice were fasted for 6 h before glucose tolerance (GTT) and insulin resistance (ITT) tests. In GTT, non-anesthetized mice were injected i.p. glucose (2 g/Kg; #G8270, Sigma Aldrich), and in the ITT, mice were injected i.p. 0.75 U/Kg of insulin (#HI0210, Lilly), prepared at 0.1 U/ml in advance. In both test, fasting plasma glucose levels were determined at time 0 and after injection of glucose (at 15, 30, 60, and 120 min) or insulin (at 30, 60, and 120 min). Glucose level was determined by glucometer (Accu-Chek Performa) in blood from tail vein at specified time points.

### Cell lines and culture conditions

Human osteosarcoma U2OS cell and their green fluorescent protein (GFP)-LC3-expressing derivatives (gift from Pr. J. Yuan, Harvard University) and HeLa cells were cultured in DMEM medium (#41966-02, Thermo Fisher Scientific) supplemented with 10% (v/v) fetal bovine serum (FBS; #F7524, Sigma Aldrich), 100 mg/L sodium pyruvate (#11360070, Thermo Fisher Scientific), 10 mM HEPES buffer (#15630080, Thermo Fisher Scientific), 100 UI/ml penicillin G sodium salt, 100 μg/mL streptomycin sulfate (#15140122, Thermo Fisher Scientific) and, for the (GFP)-LC3-expressing derivatives cell, 500 µg/mL geneticin (G418 sulfate) (#10131-27, Thermo Fisher Scientific). MEFs (mouse embryonic fibroblast) cells were cultured in the same DMEM with additional supplementation of 1 mM nonessential amino acids (#11140-035, Thermo Fisher Scientific). Human liver cancer cell line HepG2 cell was cultured in EMEM medium (#30-2003, ATCC) supplemented with 10% (v/v) FBS, 100 mg/L sodium pyruvate, 10 mM HEPES buffer, 100 UI/ml penicillin G sodium salt, 100 μg/mL streptomycin sulfate. Human colorectal cancer HCT116 WT or EP300 knockout (KO) (Cancer Technology, upon MTA:008927) were cultured in McCoy medium (#16600-082, Thermo Fisher Scientific) supplemented with 10% (v/v) FBS, 100 mg/L sodium pyruvate, 10 mM HEPES buffer, and 100 UI/mL penicillin G sodium salt, 100 μg/mL streptomycin sulfate. Murine methylcholanthrene induced fibrosarcoma MCA205 cells (class I MHC haplotype H-2b, syngeneic for C57BL/6 mice), autophagy-partially deficient (*Atg5*^*KD*^) MCA205 cells and CD39 overexpressing MCA205 cells, were cultured in RPMI-1640 medium (#61870010, Thermo Fisher Scientific) supplemented with 10% (v/v) FBS, 100 UI/mL penicillin G sodium salt, 100 μg/mL streptomycin sulfate, 1 mM sodium pyruvate and 1 mM HEPES buffer. All cells were maintained in standard culture conditions (at 37 °C, under 5% CO_2_). Cells were seeded in 6-well or 384-well plates and grown for 24 h before treatment with 5 mM sodium salicylate (#S3007, Sigma Aldrich), 3 μM C646 (#SML0002, Sigma Aldrich), 100 μM anacardic acid (#A7236, Sigma Aldrich) or 50 μM Trichostatin A (TSA) (#T8552, Sigma Aldrich) for 4, 6, or 16 h, as reported in the different settings, and then subjected to immunoblotting analysis or automated microscopy detection of GFP-LC3 dots surface or acetyl-lysine fluorescence intensity.

### Preparation of leukocytes and plasma

After blood drawing, 500 μL total blood were diluted in 5 mL red blood cell lysis buffer (#420301, BioLegend) for 10 min at room temperature and washed twice in PBS. For immunoblotting, white cells were lysed in 50 μL radio immunoprecipitation assay (RIPA) buffer. For cytofluorimetric assays, cells were fixed in 4% formaldehyde solution and processed as described below. Alternatively, 500 μL total blood were centrifuged for 10 min at 15,000 rpm, and then plasma was separated and subjected to metabolomics analyses.

### Immunoblotting

For immunoblotting, proteins extracted from isolated leukocytes obtained by cellular lysis in RIPA buffer were run on 4–12% Bis-Tris acrylamide gels (#NP0322, Thermo Fisher Scientific) and electrotransferred to 0.2 μM polyvinylidene fluoride (PVDF) membranes (#1620177, Bio-Rad). Non-specific binding sites were saturated by incubating membranes for 1 h in 0.05% Tween 20 (#P9416, Sigma Aldrich) v-v in Tris-buffered saline (TBS) (#ET220, Euromedex) supplemented with 5% non-fat powdered milk (w:v in TBS), followed by an overnight incubation with primary antibodies specific for LC3B (#2775, Cell Signaling Technology). Equal protein loading was monitored with actin specific anti-beta Actin antibody (#ab49900 [AC-15] (HRP), Abcam). Membranes were cut in order to allow simultaneous detection of different molecular weight proteins. Membranes were developed with suitable horseradish peroxidase conjugates followed by chemiluminescence-based detection with the Amersham ECL Prime (#RPN2232, GE Healthcare) and the ImageQuant LAS 4000 software-assisted imager (GE Healthcare, Piscataway, NJ, USA). Quantification was performed by densitometry by means of Image J software. Autophagy was quantified through evaluation of LC3-II/actin ratio as described in^28^.

### Immunofluorescence staining on leukocytes

For LC3 dots detection, isolated leukocytes were fixed in 4% formaldehyde solution for 20 min at 37 °C. Fixed cells were permeabilized by means of 90% methanol in water, and non-specific sites were blocked with 2% bovine serum albumin (BSA) in PBS. Cells were then incubated overnight with an anti-LC3B antibody (1:300) (#2775, Cell Signaling Technology) in 2% BSA/PBS, followed by incubation of 1 h at room temperature with Alexa-Fluor 488 anti-rabbit conjugated secondary antibody (1:300) (#A11008, Invitrogen) in 2% BSA/PBS and Hoechst 33342 (#H3570, Invitrogen). For acetylation measurements, leukocytes were fixed with 4% formaldehyde for 20 min at room temperature, blocked with 2% bovine serum albumin in PBS and incubated with anti-acetylated lysine conjugated antibody PE/Cy7 anti-mouse (#clone 15G10, 623408 BioLegend) for 1 h at room temperature. Leukocyte immunophenotyping was obtained through staining of human and murine white blood cells with anti-Alexa Fluor 647 anti-mouse PTPRC/CD45 antibody (#clone 30-F11, BioLegend) or Alexa Fluor 647 anti-human PTPRC/CD45 antibody (#clone HI30, BioLegend).

### Cytofluorometric analysis

Multispectral imaging flow cytometry was performed on an AMNIS ImageStream X Mark II equipped with 375-, 488-, 561-, and 642-nm lasers using the 60x magnification lens. Only Hoechst^+^ events were recorded. At least 10,000 cells/sample were acquired for each sample. The analysis was done with IDEAS software v6.2. Only focused events were included in the analysis, using the gradient RMS feature of bright field images. A compensation matrix was calculated using single color fluorescent control files. This matrix was applied to each file. Singlets were then gated on aspect ratio vs area of bright field and leukocyte subpopulations were identified and gated on a pictogram based on the intensity of PTPRC/CD45 staining versus dark field. The intensity of acetyl-lysine staining was quantified within the entire cell. The Spot Wizard in the IDEAS^®^ software was used to calculate the number of LC3 spots. Flow cytometric analysis of acetylation was performed on an Attune Ntx Flow Cytometer (Thermo Fisher Scientific) equipped with 405- and 488-nm lasers.

### Quantitative label-free proteomics method and analysis

#### Cell culture sample preparation

Two million HCT116 WT and EP300 KO cells were seeded in Petri dish (15 cm of diameter), grown for 4 days before treatment with 5 mM sodium salicylate for 6 h, and then collected by scrape in PBS, obtained between 15 and 20 mg of protein per sample. The cell pellet was resuspended in 5 ml of urea 8 M, and sonicated 3 × 15 s, power 30% and centrifuged at 80,000 *g* for 20 min at room temperature.

#### Protein digestion

Cell lysates (20 mg) were diluted in 8 M urea in 200 mM ammonium bicarbonate (ABC) followed by protein reduction with 5 mM DTT for 1 h at 37 °C. Reduced lysates were alkylated with 10 mM iodoacetamide for 30 min at room temperature in the dark. The samples were diluted in 200 mM ABC to reach a final concentration of 1 M urea and digested overnight at 37 °C with Trypsin (CAT#: LS003750, Worthington) at a ratio of 1/50. Digested peptide lysates were acidified with FA 50% to a final concentration of 5% FA. Samples were centrifuged at 4000 rpm and desalted on a SEP PAK C18 cartridge (CAT#: WAT051910, Waters). Peptides were eluted with 40% acetonitrile in 0.1% FA, dried under vacuum (10% was dried apart for proteomes analyses), and stored at −20 °C.

#### Immunoprecipitation

Immunoprecipitation was performed by the Cell Signaling kit (#13416S, PTM scan acetyl-lysine motif), as indicated by the manufacturer’s instructions, adjusting the pH at 6 with Tris 1.5 M. Peptides were eluted from beads with 0.15% TFA, desalted over tips packed with Empore™ C18 Extraction Disks (3 M™ Discs 2215) and eluted with 40% acetonitrile in 0.1% FA. Eluted peptides were dried under vacuum and reconstituted in injection buffer (2% acetonitrile in 0.3% TFA, trifluoroacetic acid) before nano-LC-MS/MS analysis.

#### LC-MS/MS analysis

For proteome and acetylome analyses, LC was performed with an RSLCnano system (Ultimate 3000, Thermo Scientific) coupled online to a Q Exactive HF-X mass spectrometer (Thermo Scientific). Peptides were trapped onto a C18 column (75 μm inner diameter × 2 cm; nanoViper Acclaim PepMapTM 100, Thermo Scientific) with buffer A (2/98 MeCN/H2O in 0.1% FA) at a flow rate of 2,5 µL/min over 4 min. Separation was then performed on a 50 cm × 75 μm C18 column (nanoViper Acclaim PepMapTM RSLC, 2 μm, 100 Å, Thermo Scientific) regulated to a temperature of 50 °C with a linear gradient of 2–35% buffer B (100% MeCN in 0.1% FA) at a flow rate of 300 nL/min over 211 min for proteomes and a linear gradient of 2–30% buffer B at the same flow rate over 91 min for acetylomes. MS full scans were performed in the ultra-high-field Orbitrap mass analyzer in ranges *m/z* 375–1500 with a resolution of 120,000 (at 200 *m/z*). The top 20 most intense ions were subjected to the Orbitrap for further fragmentation via HCD activation and a resolution of 15,000 with the AGC target set to 105 ions. We selected ions with charge state from 2+ to 6+ for screening. NCE was set to 27 and the dynamic exclusion of 40 s for proteomes and 20 s for acetylomes.

#### Data analysis

For identification, the data were searched against the *Homo sapiens* (UP000005640) Uni-Prot database using Sequest-HT through Proteome Discoverer (version 2.2). Enzyme specificity was set to trypsin (full) and a maximum of two miss cleavage sites were allowed for proteomes and three for acetylomes. Oxidized methionine, Carbamidomethyl cysteines and N-terminal acetylation were set as variable modifications and acetyl on lysine was added for the acetylome analyses. For both experiments, maximum allowed mass deviation was set to 10 ppm for monoisotopic precursor ions, and 0.02 Da for MS/MS peaks. The resulting files were further processed using myProMS v3.9^29^. FDR calculation used Percolator^30^ and was set to 1% at the peptide level for the whole study. The label-free quantification was performed by peptide Extracted Ion Chromatograms (XICs), computed with MassChroQ version 2.2.1^31^. For proteomes and acetylomes quantification, XICs from proteotypic peptides between compared conditions (TopN matching for proteomes) with missed cleavages were used. Median and scale normalization was applied on the total signal to correct the XICs for each biological replicate (*N* = 3). To estimate the significance of the change in protein abundance, a linear model (adjusted on peptides and biological replicates) based on a two-tailed *T* test was performed and *p*-values were adjusted with the Benjamini–Hochberg FDR procedure. Proteins with at least three total peptides in all replicates, a 2-fold enrichment and an adjusted *p* value < 0.05 were considered significantly enriched in sample comparisons. The mass spectrometry proteomics data have been deposited to the ProteomeXchange Consortium via a PRIDE^32^ repository identified with the PXD020556 (Username: reviewer50560@ebi.ac.uk, Password: i38Y9I3B).

### RNA interference in cell culture, fluorescence microscopy, and analysis

Human osteosarcoma U2OS cells stably expressing GFP-LC3 were transfected with twenty-three different small interfering RNA (siRNA) sequences targeting genes down- and up-regulated in both HCT116 EP300 WT cells and in HCT116 EP300 KO cells treated with salicylate, by means of Lipofectamine RNAi MAX (#13778030, Thermo Fisher Scientific) transfection reagent. siRNAs used in this study were: αENOLASE (#sc-35310, Santa Cruz), ANP32B (#SI02655380, Qiagene), ARFGAP3 (#sc-60200, Santa Cruz), BAG6 (#sc-72614, Santa Cruz), CASP8AP (#EHU044071, Sigma Aldrich), EP400 (#EHU099591, Sigma Aldrich), HIST1H2BM (#EHU112351, Sigma Aldrich), HMBS (#EHU058501, Sigma Aldrich), HMGA1 (#EHU122721, Sigma Aldrich), HNRNPU (#SI02781002, Qiagene), HSP90AB1 (#SI03055304, Qiagene), KRT8 (#EHU112091, Sigma Aldrich), LASP1 (#SI02654855, Qiagene), La/SSAB (#sc-40915, Santa Cruz), MALM1 (#EHU043911, Sigma Aldrich), P4HB (#SI02662639, Qiagene), PDIA4 (#EHU049031, Sigma Aldrich), PTMS (#EHU142221, Sigma Aldrich), SKIV2L2 (#EHU010001, Sigma Aldrich), STARD9 (#EHU039181, Sigma Aldrich), TCOF1 (#sc-61707, Santa Cruz), USP7 (#EHU131171, Sigma Aldrich), ZNF302 (#EHU115041, Sigma Aldrich). U2OS GFP-LC3 cells were seeded in 384-wells imaging plates (BD Falcon, Sparks, USA), transfected with the indicated siRNA and after 24 h, treated for 16 h with 5 mM sodium salicylate in absence or presence of the lysosomal inhibitor, 100 nM bafilomycin A1 for the last 2 h of treatment. Subsequently, cells were fixed with 4% formaldehyde solution and counterstained with 10 μM Hoechst 33342. Images were acquired using an automated fluorescence microscopy, a robot-assisted Molecular Devices IXM XL BioImager (Molecular Devices, Sunnyvale, CA, USA) equipped with either a SpectraX or an Aura II light source (Lumencor Beaverton, OR, USA), adequate excitation and emission filters (Semrock, Rochester, NY, USA) and a 16-bit monochromes sCMOS PCO.edge 5.5 camera (PCO Kelheim, Germany) or an Andor Zyla camera (Belfast, Northern Ireland), and a ×20 PlanAPO objective (Nikon, Tokyo, Japan) were used to acquire a four view fields per well, followed by automated image processing with the custom module editor within the MetaXpress software (Molecular Devices). Image segmentation was performed using the MetaXpress software (Molecular Devices). The primary region of interest (ROI) was defined by a polygon mask around the nucleus allowing for the enumeration of cells, and the quantification of nuclear area and nuclear fluorescence intensity. For the quantification of the surface of LC3 dots, a mask of high-intensity dots was drawn in the cytoplasm of cells. Cellular debris and dead cells (low nuclear area, Hoechst^high^) were excluded from the data set, and the surface of LC3 dots was normalized on control, statistically evaluated, and graphically depicted using R software.

### Immunofluorescence staining and analysis

For the cytoplasmic acetyl-lysine staining, U2OS WT or HCT116 WT and EP300 KO cells were fixed with 4% formaldehyde solution for 20 min at room temperature. After PBS wash, block of non-specific binding sites and cell permeabilization were done with anti-acetylated-tubulin antibody (1:400) (#5335 S, Ozyme) in 5% BSA/PBS, followed by incubation with primary antibody anti-acetylated-Lys (1:100) (#BLE-623402, Biolegend) in 2% BSA/PBS, overnight at 4 °C. Following the cells were incubated, for 1 h at room temperature, with secondary antibody (1:300) Alexa Fluor 568 anti-mouse (#A11031, Invitrogen). 10 μM Hoechst 33342 (#H3570, Invitrogen) was employed for nuclear counterstaining. To determine the acetyl-lysine fluorescence intensity, the BD pathway 855 automated microscope (BD Imaging Systems, San Jose, USA) equipped with a 20X objective (Olympus, Center Valley, USA) coupled to a robotized Twister II plate handler (Caliper Life Sciences, Hopkinton, USA), was used. Images (5 × 5 frames), corresponding to 100–500 cells/well, were analyzed for the detection of acetyl-Lys intensity in the cytoplasm by means of the BD Attovision software (BD Imaging Systems). For quantitative analyses of protein acetylation, cell surfaces were segmented into cytoplasmic and nucleic region, according to standard procedures, and average staining intensity of each individual cell was measured for statistical analysis.

### Determination of extracellular ATP concentrations

Extracellular ATP levels were measured by the luciferin-based ENLITEN^®^ ATP Assay (Promega, Charbonnieres, France) kit as indicated by the manufacturer’s instructions. ATP-driven chemiluminescence was recorded on a Fluostar multiwell plate lumi nometer (BMG Labtech, Offenburg, Germany).

### Statistical analysis

Prism GraphPad (SanDiego, CA, USA) software was used to perform graphs and statistical analyses, unless otherwise indicated. Data are reported as box and whisker plots, which show median, first and third quartiles, and maximum and minimum values or as means ± s.e.m., as specified. Circles indicate each mouse used in the experiment, unless otherwise stated in the manuscript. For statistical analyses, *p* values were calculated by, one-way ANOVA non-corrected, two-way ANOVA non-corrected, by two-tailed unpaired Student’s *t* test or by two-tailed paired Student’s *t* test. Longitudinal statistical comparisons, for mice weight gain and tumor growth, were performed with https://kroemerlab.shinyapps.io/TumGrowth/^33^. Wald test was used to compute *p* values, by testing jointly that both slopes and intercepts were the same between the groups of interest. Kolmogorov test was used to compare adipocytes size, by comparing their size distributions. Fisher’s exact test was used to compare convergence incidences between aspirin and NF metabolites. Chi square analysis was used to compare autophagy-relevant salicylate targets among the EP300 and the non-EP300 targets. Differences were considered statistically significant when *p* values are: *(*p* < 0.05), **(*p* < 0.01), ***(*p* < 0.001) and *(*p* < 0.01) for the Fisher’s exact test.

## Results

### Comparison of aspirin vs fasting effects in mice

**Fig. 1:**
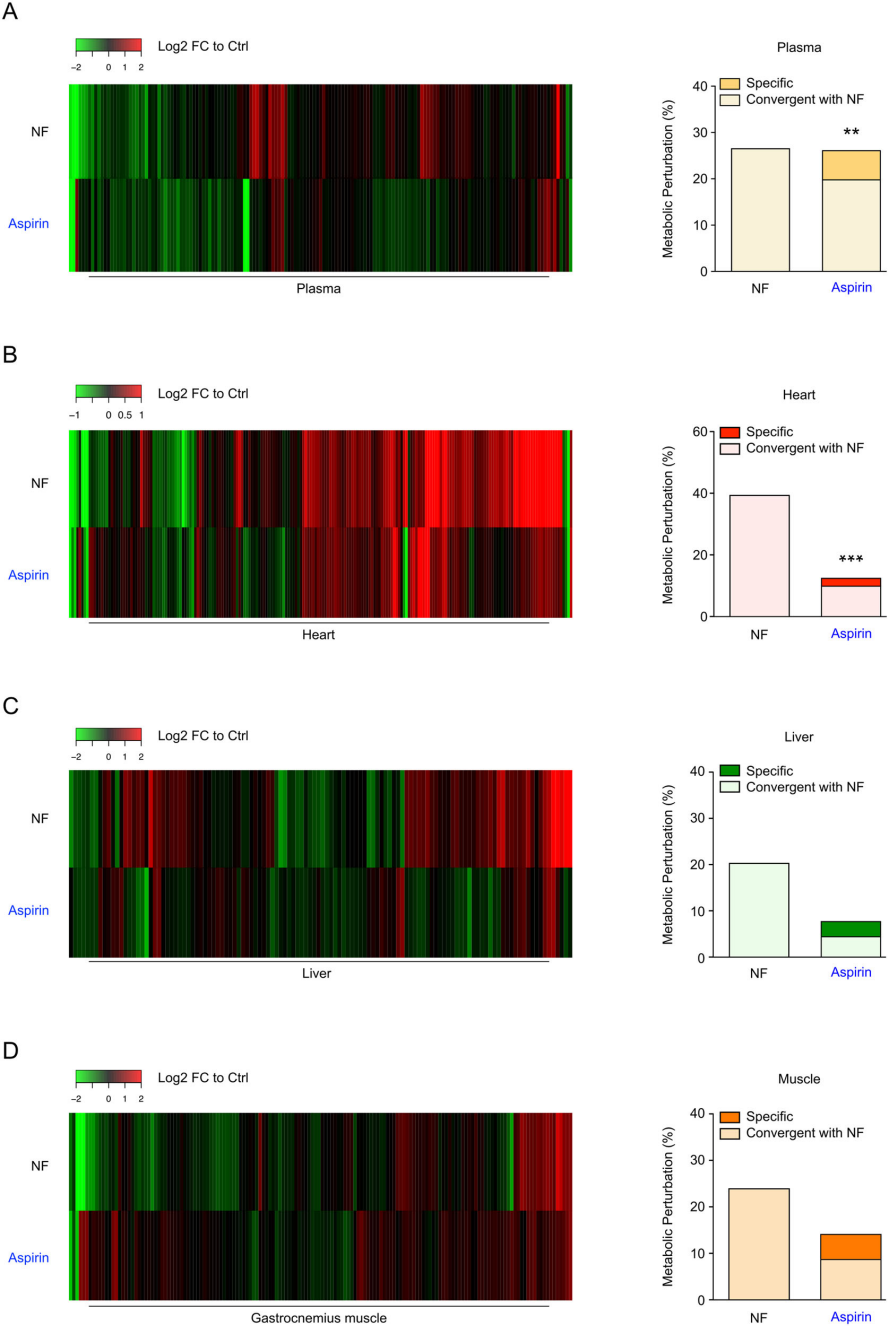
Metabolic changes induced by aspirin administration and fasting in mice. **a**–**d** WT male mice were subjected to 48 h of starvation or treated with aspirin 100 mg/kg i.p. for 6 h, then blood was drawn, and metabolic analyses were performed on plasma (**a**), heart (**b**), liver (**c**) and gastrocnemius muscle (**d**) (for Ctrl *n* = 10 mice; starvation *n* = 5 mice; aspirin *n* = 5 mice). Heat maps (left panels) depict Log2 Fold Change (FC) values to control mice (False Discovery Rate < 0.1) for annotated and putative metabolites (listed in Supplemental Table 1). Bar plots (right panels) display the percentage of metabolic perturbation (i.e. metabolites that are significantly changed in treated groups (*p* < 0.05) compared to Ctrl animals). Aspirin-induced alterations were considered as convergent with starvation when they had the same sign. For statistical analyses, *p* values were calculated by Fisher’s exact test to compare convergence incidences with those in nutrient free, (**p < 0.01; ***p < 0.001). Ctrl control, FC fold change, NF nutrient free. Data associated with fasted animals are shared with (26).

The biochemical features of starvation can be partially recapitulated by so-called caloric restriction mimetics (CRMs), which are non-toxic synthetic molecules or natural compounds endowed with the capacity to reduce cellular protein acetylation, thereby igniting the autophagic reaction^34^. One agent that falls into this definition is aspirin^14^. To compare the effects evoked by aspirin medication with those induced by starvation on the (annotated and putative) metabolome, we performed mass spectrometric metabolomics analysis of mice treated with 100 mg/kg aspirin (intraperitoneally administration, i.p.) or subjected to a 48 h fasting. We found that aspirin produces metabolic variations that are significantly convergent (*p* < 0.01, Fischer’s exact test) with those elicited by starvation in the plasma (Fig. 1a and Supplemental Table 1) and in the heart (Fig. 1b and Supplemental Table 1), yet failed to achieve such effects in the liver (Fig. 1c and Supplemental Table 1) and the gastrocnemius muscle (Fig. 1d and Supplemental Table 1). Important common metabolic changes were also induced in several cell lines from human and mouse origin upon culture with salicylate (Fig. S1A). These results suggest that aspirin can induce changes similar to fasting in the extracellular compartment in vivo, while it displays an organ-specific pattern of metabolic effects at the intracellular level.

### Protein deacetylation induced by aspirin

**Fig. 2:**
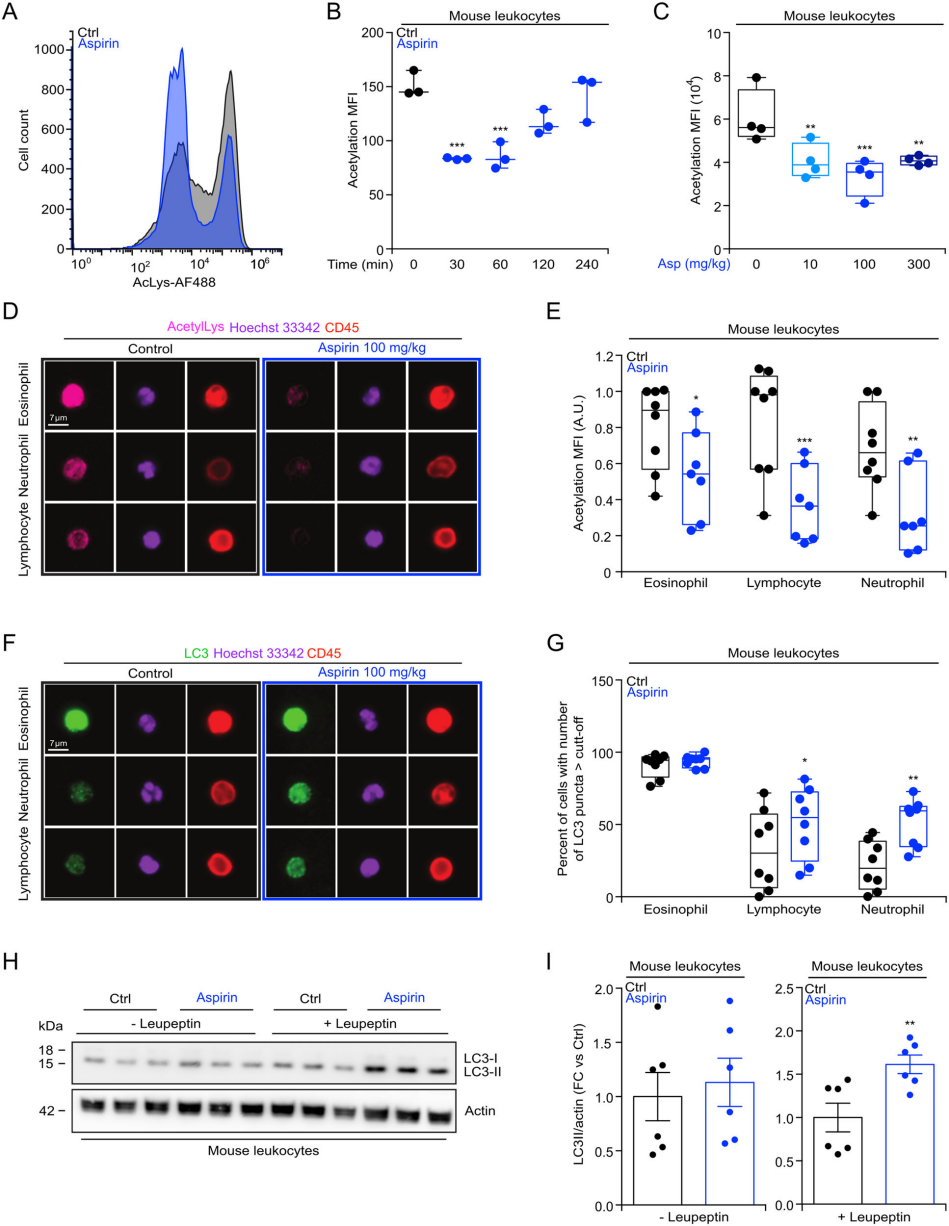
Aspirin mediated protein deacetylation and autophagy induction in mouse leukocytes. **a**–**c** WT male mice (7 weeks old) received intraperitoneally (i.p.) injections of aspirin (100 mg/kg) and blood was collected at different time points (30, 60, 120, and 240 min post aspirin administration) (**b**), or mice were treated with different dose of aspirin (10, 100, or 300 mg/kg i.p. administration) and blood collected after 1 h (**c**), to determine the level of Nε lysine acetylation (representative image in (**a**), quantification in (**b**, **c**) (*n* = 3/4 mice/group). **d**–**g** WT male mice were treated with aspirin (i.p. 100 mg/kg) and 6 h later, the level of Nε lysine acetylation (representative image in (**d**), quantification in (**e**) (Ctrl = 8 mice, aspirin = 7 mice) and autophagy induction, measured by counting the number of LC3B puncta per cell, in mice treated 2 h before the recovery of blood with leupeptin (i.p., 15 mg/kg), (representative image in (**f**), quantification in (**g**)) (*n* = 8 mice/group), were determined by immunofluorescence staining in the different leukocytes populations. **h**, **i** In the same experimental conditions described in (**d**–**g**), the autophagy associated level of LC3 lipidation was evaluated in the absence or presence of leupeptin (i.p., 15 mg/kg) administered 2 h before the recovery of blood (representative blots in (**h**), quantitation in (**I**)) (*n* = 3 mice/group, 2 independent experiments). In this figure, data are displayed as box and whisker plots, which show median, first and third quartiles, and maximum and minimum values (**b**, **c**, **e**, **g**) or as scatter dot plot as mean ± s.e.m. **i** Circles, in the graphs, indicate each mouse used in the experiment. Statistical comparisons were done by one-way ANOVA (**b**, **c**), two-way ANOVA (**e**, **g**) and two-tailed unpaired Student’s *t* test (**i**) comparing aspirin-treated to untreated mice (*p < 0.05, **p < 0.01, ***p < 0.001). A.U arbitrary unit, Ctrl control, FC fold change, MFI mean fluorescence intensity, min minute.

Next, we investigated the effects of aspirin on mouse leukocytes with respect to protein lysine acetylation in vivo. Circulating white blood cells from i.p. injected mice (Fig. 2a, b) manifested a rapid (30–60 min) and transient reduction of protein acetylation, detectable by immunofluorescence staining with an antibody recognizing ε-acetylated lysine residues. This effect was dose-dependent, requiring at least 10 mg/kg body weight to induce protein deacetylation in mice (Fig. 2c). Moreover, a dose of 100 mg/kg of aspirin is able to induce protein deacetylation after 6 h of treatment, in all mouse leukocyte subsets (eosinophils, lymphocytes, and neutrophils) (Fig. 2d, e). Deacetylation of Nε lysine residues of cytoplasmic proteins can stimulate autophagy^35^, and a significant autophagy induction is detected in circulating leukocytes (except for eosinophils), as revealed by immunofluorescence staining and immunoblotting (Fig. 2f–i). These results demonstrate that aspirin induces protein deacetylation and autophagy in circulating immune cells.

### Aspirin-induced deacetylation beyond EP300 inhibition

**Fig. 3:**
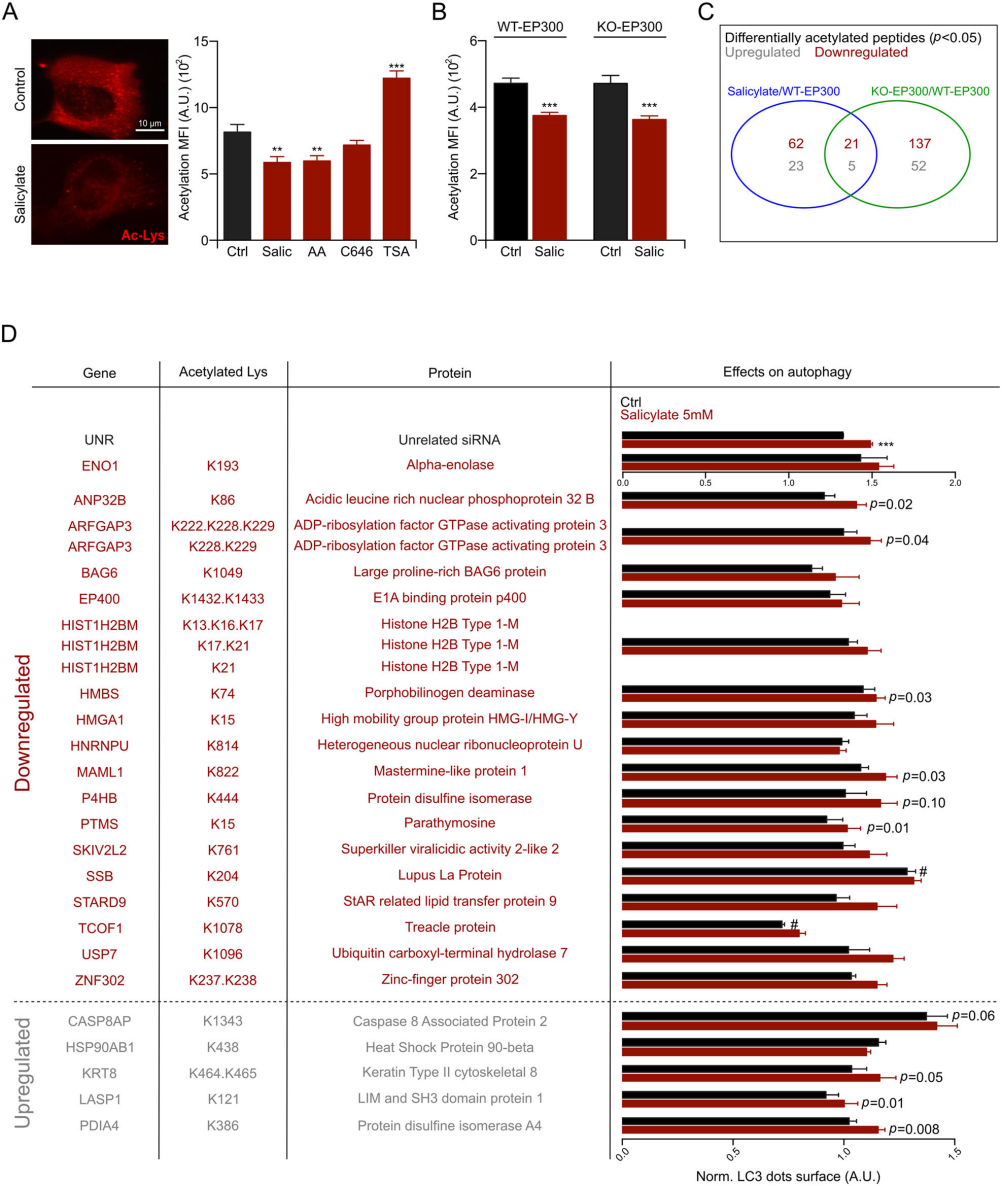
Aspirin induces deacetylation beyond EP300 inhibition. **a** U2OS WT cells were seeded in 384-wells plate and treated with 5 mM sodium salicylate (Salic), 100 μM anacardic acid (AA), 3 μM C646 or 50 μM trichostatin A (TSA) for 4 h and then subjected to immunofluorescence for the determination of Nε lysine acetylation level (representative image on the left and quantification on the right). **b** HCT116 WT and EP300 KO cells were treated with 5 mM Salic for 6 h and Nε lysine acetylation level was determined. **c** Venn diagram representing acetylated peptide up- or downregulated in HCT116 WT cells treated with salicylate or in HCT116 EP300 KO cells. **d** U2OS GFP-LC3-expressing cells were transfected with siRNA of the up- or downregulated protein identified in (**c**), and then treated with 5 mM Salic for 16 h, in the presence of the lysosomal inhibitor bafilomycin A1 (100 nM) for the last 2 h of treatment, followed by quantitation of GFP-LC3 dots surface. The results were normalized on the control (Ctrl) unrelated (UNR) siRNA. Effects of siRNA on basal autophagy and effects of Salic-induced autophagy, in each siRNA, are depicted in the table. Results in this figure are represented as means ± s.e.m. **a**, **b** and as means ± s.e.m. of at least three replicates (with at least 6/8 wells for each conditions) in (**d**). Statistical comparisons were performed as one-way ANOVA compared to Ctrl group (**p < 0.01, ***p < 0.001) (**a**), two-tailed unpaired Student’s *t* test (***p < 0.001) (**b**), or as *p*aired Student’s *t* test (**d**), comparing salicylate-*t*reated to untreated group (***p < 0.001) and comparing Ctrl UNR DMEM *versus* Ctrl DMEM of each siRNA (^#^*p* < 0.05) (*p* values are listed in Supplementary Table 2). AA anacardic acid, A.U arbitrary unit, Ctrl control, MFI mean fluorescence intensity, Salic sodium salicylate, TSA trichostatin A.

Aspirin induces autophagy through the inhibition of EP300^14–16^. However, inhibition of EP300 with its pharmacological antagonist C646 is not sufficient to induce protein deacetylation in cultured cells, contrasting with salicylate that significantly reduces acetylation (Fig. 3a). Similarly, EP300 KO cells manifest a normal acetylation level, comparable to that of wild-type (WT) cells, and both WT cells and EP300 KO cells underwent protein deacetylation when they were treated with salicylate (Fig. 3b). Hence, aspirin must mediate its effects on protein acetylation through EP300-independent effects, most likely through the inhibition of other acetyltransferases. To investigate this possibility, we performed a quantitative label-free enriched acetylome analysis, searching for proteins that are deacetylated upon EP300 knockout and/or salicylate treatment. This procedure led to the identification of 62 proteins that were deacetylated in salicylate-treated cells, yet apparently were not EP300 substrates. In addition, twenty-one proteins that were deacetylated in response to salicylate were also deacetylated in EP300 KO cells, suggesting that they are indeed EP300 substrates (Figs. 3c, d and Supplemental Table 2). Among these 21 proteins, 6 have previously been shown to regulate autophagy. This applies to ADP-ribosylation factor GTPase activating protein 3 (ArfGAP3)^36^, large proline-rich BAG6 protein (BAG6) (also called BAT3)^37^, high mobility group protein HMG-I/HMG-Y (HMGA1)^38^, Sjögren syndrome type B antigen, best known as Lupus La protein (SSB)^39^, StAR related lipid transfer protein 9 (STARD9)^40^ and ubiquitin carboxyl-terminal hydrolase 7 (USP7)^41^. We knocked down each of these genes (21 downregulated and 5 upregulated) and determined their effects on autophagy in the presence and absence of salicylate. Of note, the knockdown of SSB and that of CASP8AP (caspase 8 associated protein 2), whose acetylation is respectively decreased and increased, enhanced autophagy and this was not further increased by salicylate (Fig. 3d and S2A, S2B). In sum, it appears plausible that aspirin induces autophagy through actions on several autophagy relevant EP300 substrates. However, aspirin has effects on protein acetylation that are independent of EP300.

### Autophagy-dependent anti-obesity and antidiabetic aspirin effects

**Fig. 4:**
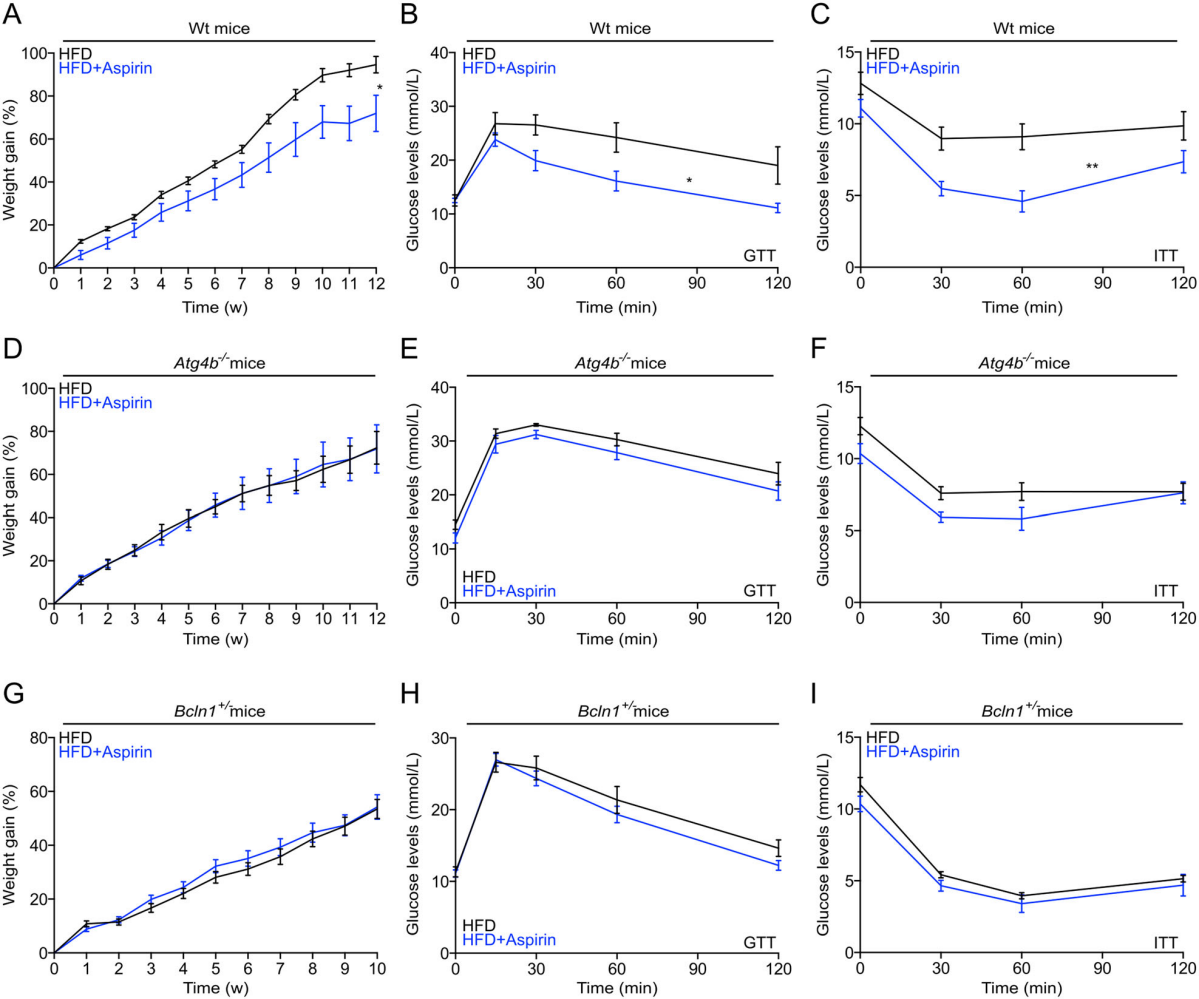
Autophagy-dependent effects of aspirin on weight gain in high-fat treated mice. **a**, **d**, **g** Male mice with the indicated genotypes (WT, *Atg4b*^−/−^ or *Bcln1*^+/−^) received high-fat diet (HFD) (WT mice: *n* = 10 mice/group; *Atg4b*^−/−^ mice: Ctrl = 8 and aspirin = 9 mice; *Bcln1*^+/−^ mice: Ctrl = 17 and aspirin = 20 mice) for at least 10 weeks in presence, or not, of daily gavage aspirin administration (100 mg/kg in EBSS). Body weight was monitored weekly, and glucose (**b**, **e**, **h**) or insulin (**c**, **f**, **i**) tolerance tests were performed after 6–11 weeks of treatment (WT mice: GTT Ctrl = 7 and aspirin = 9 mice, ITT Ctrl = 7 and aspirin = 10 mice; *Atg4b*^−/−^ mice: GTT and ITT Ctrl = 7 and aspirin = 9 mice; *Bcln1*^+/−^ mice: GTT Ctrl = 14 and aspirin = 16 mice, ITT Ctrl = 14 and aspirin = 14 mice). For statistical analysis, longitudinal statistical comparisons for mice weight gain, were performed by Wald test (**a**, **d**, **g**) (*p < 0.05); *p* values were determined by two-tailed unpaired Student’s *t* test (for **b**, **e**, **h** and **c**, **f**, **i** as areas under the curve (AUC) in Fig. S3A–S3C**)** comparing aspirin-treated to untreated mice, (*p < 0.05, **p < 0.01). Atg4b auto*p*hagy-related protein 4 homolog B, Bcln1 Beclin 1, GTT glucose tolerance test, HFD high-fat diet, min minutes, ITT insulin tolerance test, w weeks.

**Fig. 5:**
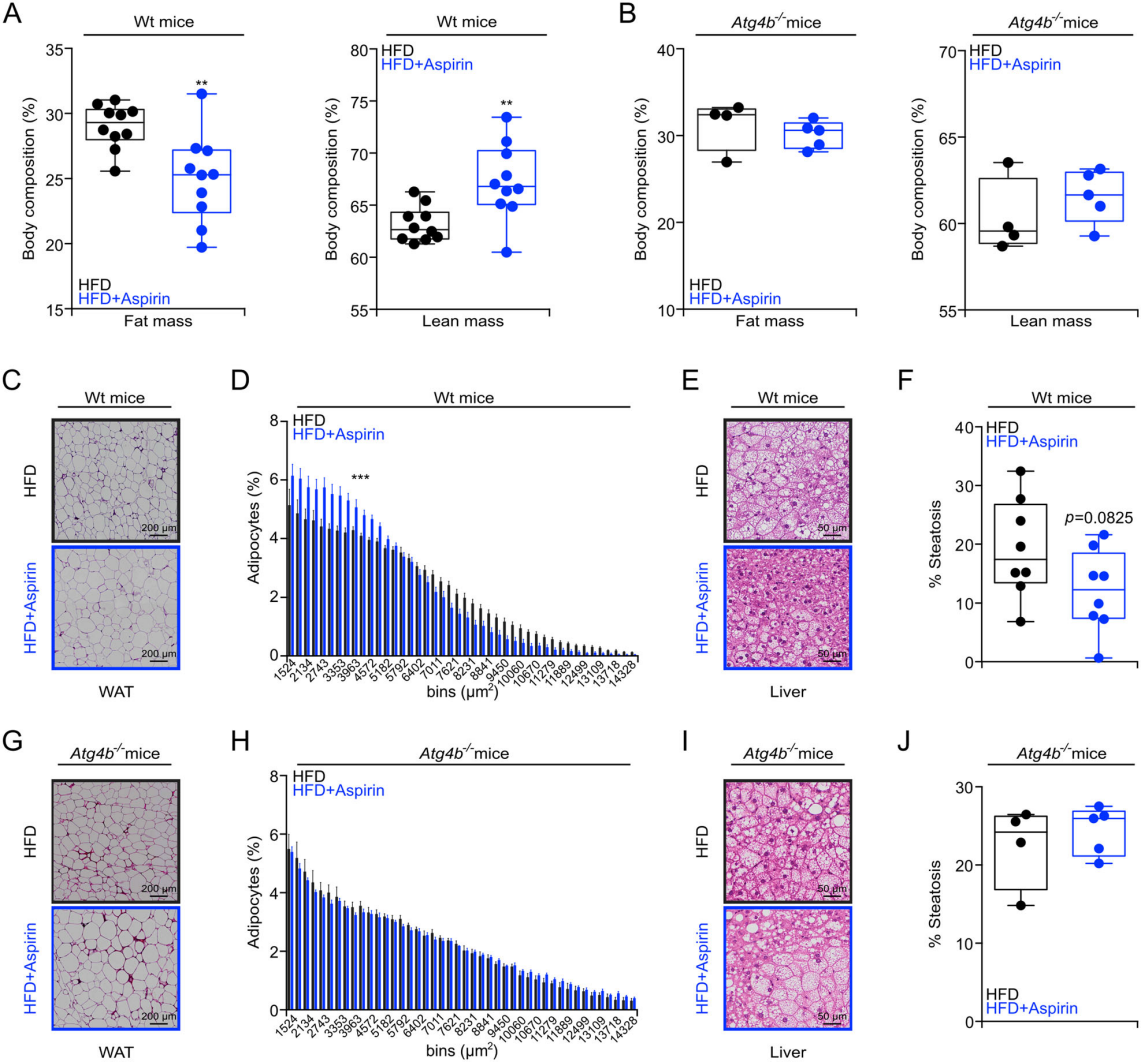
Aspirin improves metabolic outcome in obesogenic conditions in autophagy-dependent manner. **a**, **b** Respectively, WT and *Atg4b*^−/−^ male mice, at 10/12 weeks of aspirin treatment were subject to body composition analysis, determined by magnetic resonance imaging (WT mice: *n* = 10 mice/group; *Atg4b*^−/−^ mice: Ctrl = 4 and aspirin = 5 mice). **c**–**j** Histological analysis of visceral white adipose tissue (**c**, **d**, **g**, **h**), liver histopathology (**e**, **f**, **i**, **j**) were performed and quantified in WT mice (visceral white adipose tissue analysis: Ctrl = 9 and aspirin = 10 mice; liver histopathology: *n* = 8 mice/group) and *Atg4b*^−/−^ mice (visceral white adipose tissue analysis and liver histopathology: Ctrl = 4 and aspirin = 5 mice). Representative images are shown in (**d**, **e**, **g**, **i)** and quantifications are reported in (**d**, **f**, **h**, **j**) respectively. In this figure, results are displayed as box and whisker plots, which show median, first and third quartiles, and maximum and minimum values (**a**, **b**, **f**, **J**) or means ± s.e.m. **d**, **h** Circles, in the graphs, indicate each mouse used in the experiment. For statistical analysis, *p* values were calculated by two-tailed unpaired Student’s *t* test (**a**, **b**, **f**, **j**) comparing aspirin-treated to untreated mice (**p < 0.01). Statistical comparisons in (**d**, **h**) were done by means of a Kolmogorov–Smirnov test comparing aspirin-treated to control group (***p < 0.001). Atg4b autophagy-related protein 4 homolog B, HFD high-fat diet, WAT white adipose tissues.

Aspirin can improve metabolic syndrome in the context of high-fat diet (HFD)^8^. Accordingly, constant treatment with aspirin reduced the weight gain induced by HFD in WT mice (Fig. 4a). Moreover, mice under HFD (3 months) that were daily treated with aspirin exhibited improved glucose tolerance (Fig. 4b and S3A, left panel) and insulin responses (Fig. 4c and S3A, right panel). Importantly, the anti-obesity and antidiabetic effects of aspirin were lost when HFD-induced weight gain was investigated in the context of genetically determined autophagy deficiencies. Thus, mice lacking Atg4b (autophagy-related protein 4 homolog B) gene (genotype: *Atg4b*^−/−^), which is one among the four Atg4 proteases, and mice that are haploinsufficient for Beclin 1 (genotype: *Bcln1*^+/−^) failed to ameliorate their phenotype, glucose tolerance and insulin response upon aspirin treatment (Fig. 4d–i and S3B, S3C). Additionally, in WT mice subjected to HFD, aspirin significantly reduced fat mass and increased lean mass (Fig. 5a), decreased adipocyte size, and attenuated hepatosteatosis (Fig. 5c–f). In contrast, aspirin failed to ameliorate fat mass, visceral adiposity, and hepatosteatosis in the partially autophagy-deficient *Atg4b*^−/−^ mice (Figs. 5B, g–j). In conclusion, it appears that aspirin mediates its favorable metabolic effects in an autophagy-dependent fashion.

### Aspirin improves chemotherapy in autophagy-dependent manner

**Fig. 6:**
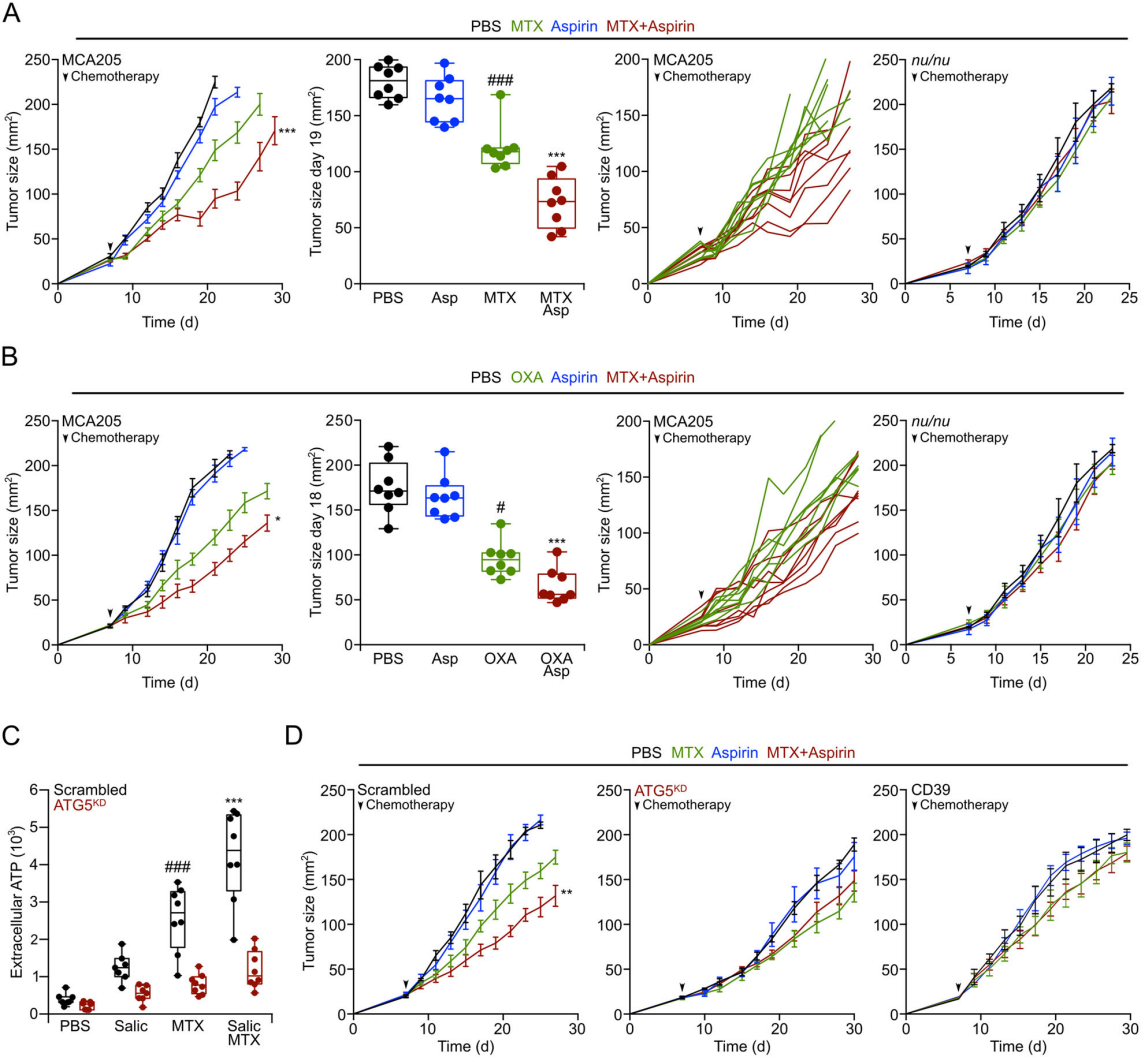
Aspirin improves chemotherapy in an immune system and autophagy-dependent fashion. **a**, **b** WT immunocompetent and athymic (*nu/nu*) mice were subcutaneously injected with MCA205 cells and when tumors became palpable, were divided into four groups and intraperitoneally treated with 5.17 mg/kg mitoxantrone (MTX) (**a**) or 10 mg/kg oxaliplatin (OXA) (**b**), alone or in combination with daily 100 mg/kg i.p. aspirin administration, or an equivalent volume of PBS. From left to right: (1) means ± s.e.m. tumor growth curves of WT mice treated with aspirin alone or in combination with MTX or OXA (*n* = 8 mice per group); (2) tumor size distribution at day 19 (MTX) or day 18 (OXA) of data shown in (1); (3) individual growth curves from mice treated with MTX or OXA alone or combined with aspirin of data shown in (1); (4) averaged ± s.e.m. tumor growth curves from immunodeficient *nu/nu* mice subjected to aspirin administration alone or in combination with MTX (*n* = 5 mice/group) or OXA (Ctrl, OXA and aspirin with *n* = 5 mice/group, OXA + aspirin *n* = 6 mice/condition). PBS and aspirin groups are shared in (**a**) and (**b**) for the *nu/nu* mice experiment. **c** Extracellular ATP levels (n = 7/8 replicates) were measured in the supernatants of autophagy competent or *Atg5*^KD^ MCA205 cells treated with 2 mM MTX and/or 5 mM sodium salicylate (Salic). **d** WT mice were inoculated subcutaneously with autophagy competent cells expressing a scrambled control shRNA (left panel) or *Atg5*^KD^ MCA205 cells (middle panels) or with CD39 overexpressing MCA205 cells (right panel). When tumors became palpable, mice were treated as in (**a**) (*n* = 8 mice/condition). Data are shown as means ± s.e.m. for tumor growth curves, as individual curves of each mice in the group, or as box and whisker plots, which show median, first and third quartiles, and maximum and minimum values. Circles in the graphs (**a**, **b**) indicate each mouse used in the experiment, in (**c**) indicate each replicate. Statistical analysis was performed by linear mixed effect modeling (over the whole-time course) and linear modeling for the single time point (*p < 0.05, **p < 0.01, ***p < 0.001 for MTX or OXA *versus* MTX + aspirin or OXA + aspirin; ^*#*^*p* < 0.05, ^###^*p* < 0.001 for PBS *versus* MTX or OXA alone). In (**c**) statistical analysis was done by two-way ANOVA Tukey’s multiple comparisons test (***p < 0.001 MTX *versus* MTX + salicylate, ^###^*p* < 0.001 PBS *versus* MTX). Asp aspirin, d day, MTX mitoxantrone, OXA oxaliplatin, Salic salicylate.

Autophagy inducers can be used to improve the efficacy of anticancer treatments using immunogenic cell death inducers such as the anthracyclines mitoxantrone (MTX) and the platinum-based compound oxaliplatin (OXA). This effect is mediated by an enhanced release of adenosine triphosphate (ATP) from tumor cells, then increasing the recruitment of immune effectors in the tumor bed due to the action of ATP on purinergic receptors^26,42–45^. For this reason, we evaluated the capacity of aspirin to improve tumor growth control by chemotherapy with MTX (Fig. 6a) or OXA (Fig. 6b) on subcutaneous MCA205 fibrosarcomas evolving on immunocompetent mice. While aspirin alone had no antineoplastic activity, it improved tumor growth reduction by MTX and OXA, and this effect was lost when the tumors developed in athymic *nu*/*nu* mice, which lack mature T lymphocytes (Fig. 6a, b, right panel**)**. Aspirin enhanced the chemotherapy-induced release of ATP from tumor cells, and this effect was lost upon knockdown of the essential autophagy gene *Atg5* (Fig. 6c). Moreover, tumor cells engineered to lack *Atg5* or to express the ATP-destroying ectoenzyme CD39 failed to respond to chemotherapy alone or in combination with aspirin even in the context of an intact immune system (Fig. 6d). Thus, aspirin acts as other autophagy inducers to improve chemotherapy-stimulated anticancer immunosurveillance.

## Discussion

Shortly after its oral uptake or injection, aspirin is metabolized to generate salicylate as well as other molecular entities that may mediate part of the pro-health effects ascribed to aspirin^14^. It will be worthy of future investigation to solve the conundrum whether metabolic oscillations elicited by aspirin in rodents are conserved in human subjects and to investigate the molecular identity of metabolites that are commonly upregulated across different species.

Aspirin produced major metabolic changes that were similar to those induced by fasting, in line with the idea that this drug acts as a caloric restriction mimetic^14^. In view of the untargeted metabolomics approach utilized in this study, in which metabolic changes are captured at the steady state, additional experiments will be required to obtain detailed insights on the metabolic pathways that are convergently intercepted by fasting and aspirin medication. ‘Fluxomics’ analyses based on the administration of non-radioactive isotope-labeled metabolites to mice would be instrumental for this task.

When administered to mice, aspirin caused a general reduction in protein deacetylation in peripheral blood mononuclear cells, as well as a significant surge in autophagy, coincident with the increase in plasma salicylate^14^. As inferable from the untargeted metabolomics analysis presented here, aspirin treatment does not seem to elicit any significant changes in the levels of metabolites (i.e. acetyl CoA, NAD) causally relevant for the reduction in protein acetylation (22). While further studies are required to confirm this experimental observation, it is nonetheless tempting to speculate that the general inhibitory action of salicylate on protein acetyltransferases is epistatic to the metabolic changes induced by the drug and hence accounts for the reported phenomena of protein deacetylation and autophagy induction.

Indeed, salicylate was able to cause protein deacetylation when added to cultured cells. This effect could be differentiated from salicylate-mediated EP300 inhibition. Thus, genetic or pharmacological inhibition of EP300 was insufficient to cause global protein deacetylation measurable by immunofluorescence technology. Moreover, salicylate could induce bulk protein deacetylation in EP300-deficient cells, and quantitative label-free proteomic experiments confirmed the capacity of salicylate to stimulate the deacetylation of 62 proteins that are not EP300 substrates. Among these proteins only six have been involved in autophagy control (ArfGAP3^36^, BAG6 (also called BAT3)^37^, HMGA1^38^, LA/SSB^39^, STARD9^40^, and USP7^41^), contrasting with the fact that among the 21 salicylate targets that are also EP300 substrates, a total of six distinct proteins is involved in autophagy. Hence, autophagy-relevant aspirin/salicylate targets are significantly (*p* < 0.05, Chi square analysis) enriched among the EP300 substrates as compared to the non-EP300 targets, confirming the functional link between EP300 inhibition by salicylate and autophagy induction.

We and others have previously demonstrated that fasting or other caloric restriction mimetics than aspirin (such as spermidine and resveratrol) also induce a broad deacetylation of multiple proteins involved in the regulation or execution of autophagy^34^. Hence, it appears that a multipronged (rather than single-molecule) deacetylation pathway explains the effects of caloric restriction mimetics with respect to autophagy induction. Aspirin limits weight gain, glucose intolerance, insulin resistance, and hepatosteatosis under HFD. Importantly, all these effects were only found in normal, autophagy-competent mice, but lost in autophagy-deficient *Atg4b*^−/−^ or *Bcln1*^+/−^ animals, strongly suggesting that they depend on autophagy. It has previously been reported that aspirin inhibits liver inflammation through the inhibition of the NF-κB activating kinase IKKβ^12,13^. There is a dual link between autophagy and IKKβ activation. On the one hand, starvation-induced autophagy is associated with IKKβ activation^46,47^, but there are also reports on an antagonistic relationship between both phenomena^48^. Hence, further exploration of the possible anti-inflammatory effects of aspirin-induced autophagy is warranted.

In mice bearing genetically determined defects in the autophagic pathway, aspirin lost its favorable effects on high-fat diet-induced metabolic syndrome. Autophagy is inhibited in human obesity because of the suppressive effects of high metabolite concentrations (in particular glucose and free fatty acids) and high levels of trophic hormones (in particular insulin and insulin growth factor)^49,50^. It remains to be seen whether such an acquired autophagic defect would ‘lock’ the morbidly obese subjects in a therapy-refractory state causing resistance against the beneficial effects of aspirin and other caloric restriction mimetics.

In sum, the experimental evidence presented here indicates that the induction of autophagy by aspirin accounts for most if not all beneficial actions of the drug. As a note of caution, it remains to be determined which organs and cell types are most responsive to autophagy induction by aspirin and whether such organs and cell types constitute preferential targets for its health-improving effects.

## Supplementary information

Legends to Supplemental figures

Figure S1

Figure S2

Figure S3

Table S1

Table S2

Table S3
