# Supplementary material for: Autophagy-mediated metabolic effects of aspirin
Source: Cell Death Discov. 2020 Nov 24;6:129. doi: 10.1038/s41420-020-00365-0 (PMC7687910; doi:10.1038/s41420-020-00365-0)
Supplement: Supplementary file 1 — Legends to Supplemental figures [file 41420_2020_365_MOESM1_ESM.docx]

**Legends to Supplemental figures**

**Figure S1. Related to Figure 1. (A)** Metabolic perturbations induced by 6 hours of 5 mM sodium salicylate treatment in human HCT116, HepG2, HeLa, U2OS and mouse MEFs cell lines. Annotated metabolites (listed in **Supplemental Table 2**) with FDR (False Discovery Rate) < 0.1 are shown.

**Figure S2. Related to Figure 3. (A-B)** Representative pictures of U2OS cells, constitutively expressing the GFP-LC3 transgene, transfected with unrelated (UNR), anti-SSB (**A**) or anti-CASP8AP (**B**) small interference RNA (siRNA); cells were left untreated (control) or were incubated with 5 mM sodium salicylate for 16 h.

**Figure S3. Related to Figure 4. (A-C)** AUC of glucose and insulin tolerance test of curves represented respectively in **Figures 4B,C, 4E,F** and **4H,I** respectively. Data are shown as mean ± s.e.m., for statistical analyses, *p* values were calculated by two-tailed unpaired Student’s *t* test comparing aspirin-treated to untreated mice (**p* < 0.05, ***p* < 0.01). Atg4b: autophagy-related protein 4 homolog B; AUC: area under the curve; Bcln1: Beclin 1; GTT: glucose tolerance test; HFD: high fat diet; ITT: insulin tolerance test.

**Table S1.** List of metabolites depicted in **Figure 1A-D** used for the analysis of convergence between starvation and aspirin treatment in different mouse organs (plasma, heart, liver and gastrocnemius muscle). Data associated to fasted animals are shared with (26).

**Table S2.** List of annotated metabolites depicted in **Figure S1**. Values refer to log2 Area (Quality Control corrected) for individual Control (Ctrl n=4) versus Aspirin (Asp n=4) conditions in different cell lines.

**Table S3.** Raw data referring to LC3 dots surface of mean of, at least, three different experiments (and respective *p* values) of data depicted in **Figure 3D**.
