# Supplementary figures and images for: Autophagy-mediated metabolic effects of aspirin
Source: Cell Death Discov. 2020 Nov 24;6:129. doi: 10.1038/s41420-020-00365-0 (PMC7687910; doi:10.1038/s41420-020-00365-0)

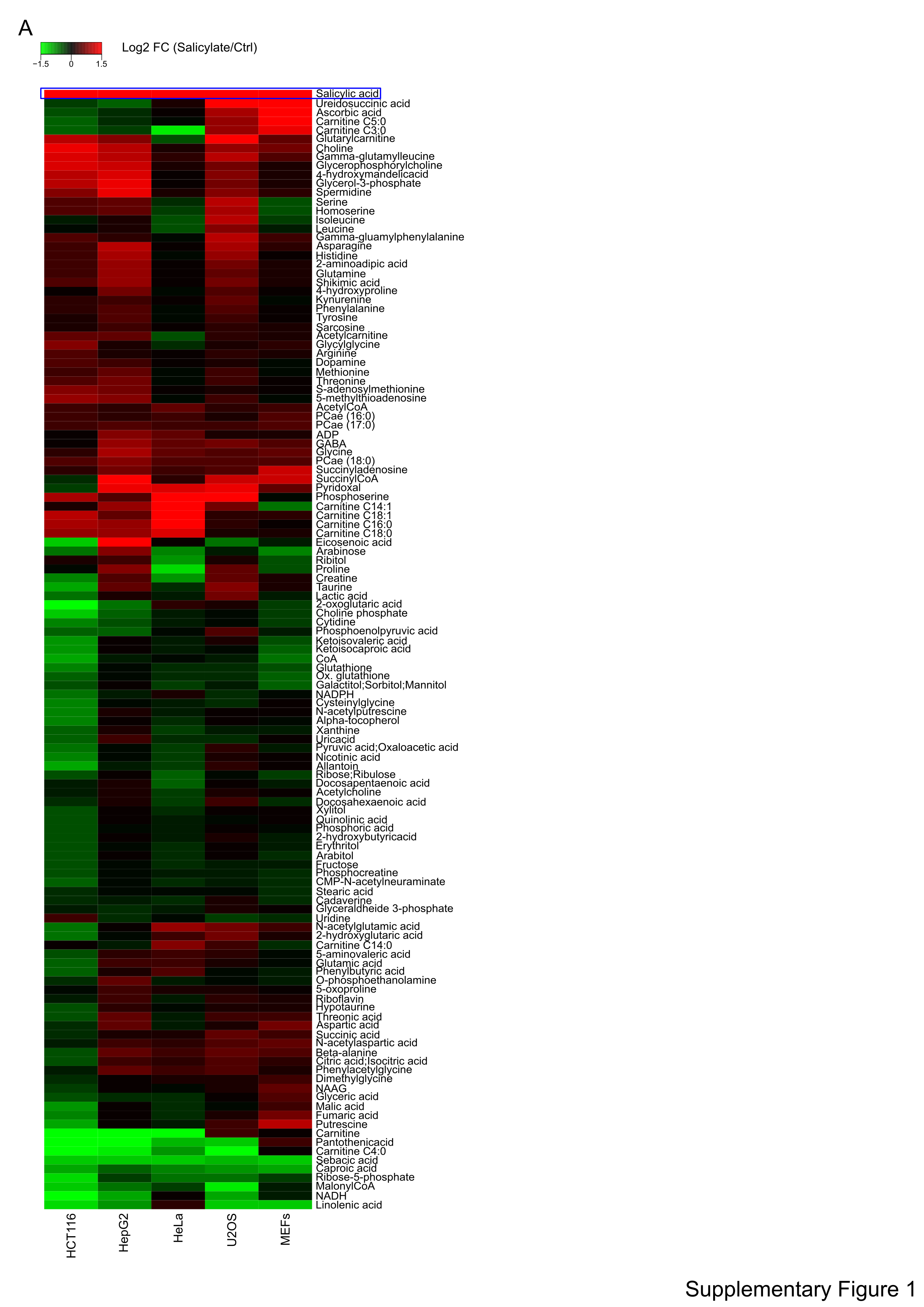

Supplement: Supplementary file 2 — Figure S1 [file 41420_2020_365_MOESM2_ESM.png]

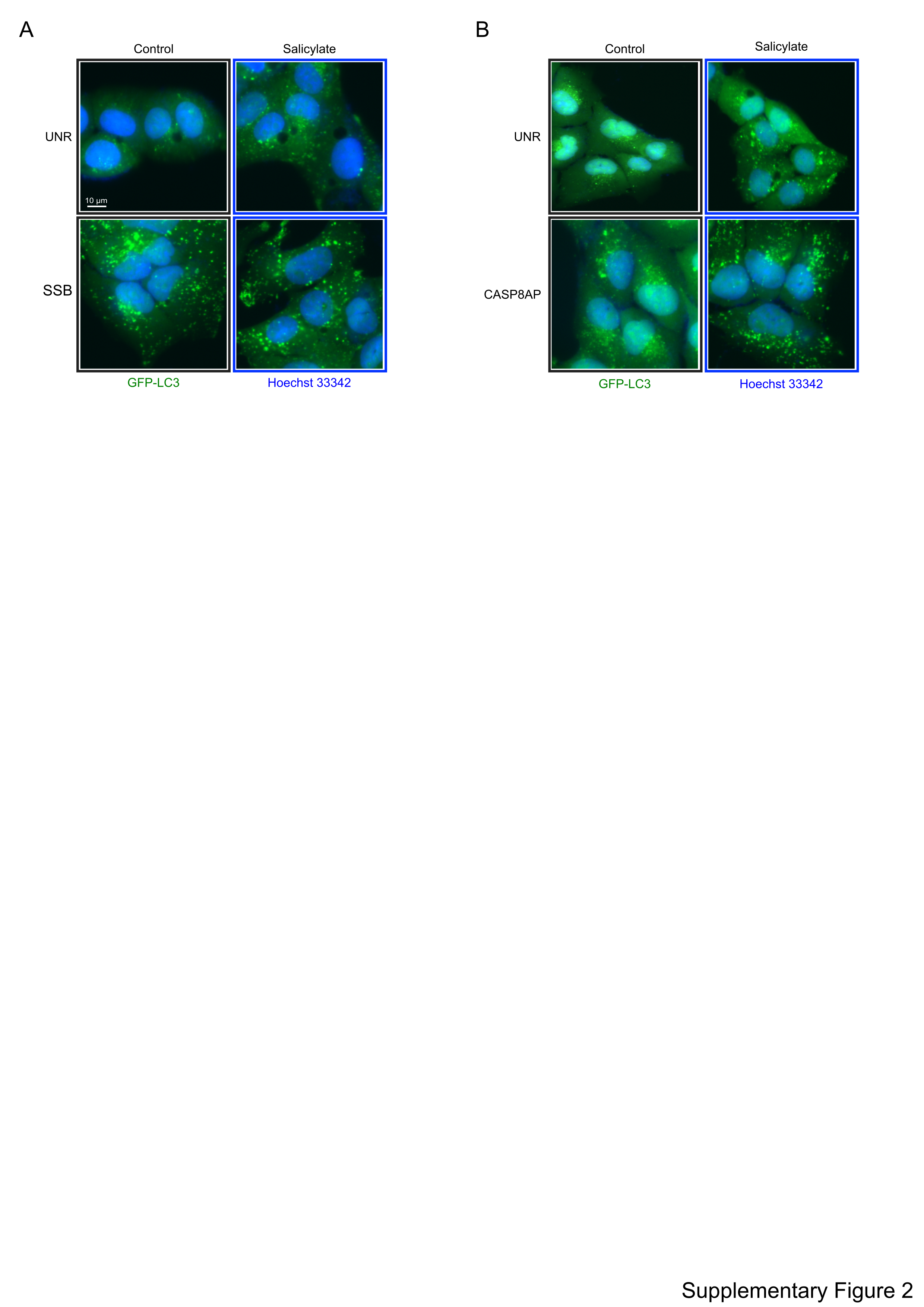

Supplement: Supplementary file 3 — Figure S2 [file 41420_2020_365_MOESM3_ESM.png]

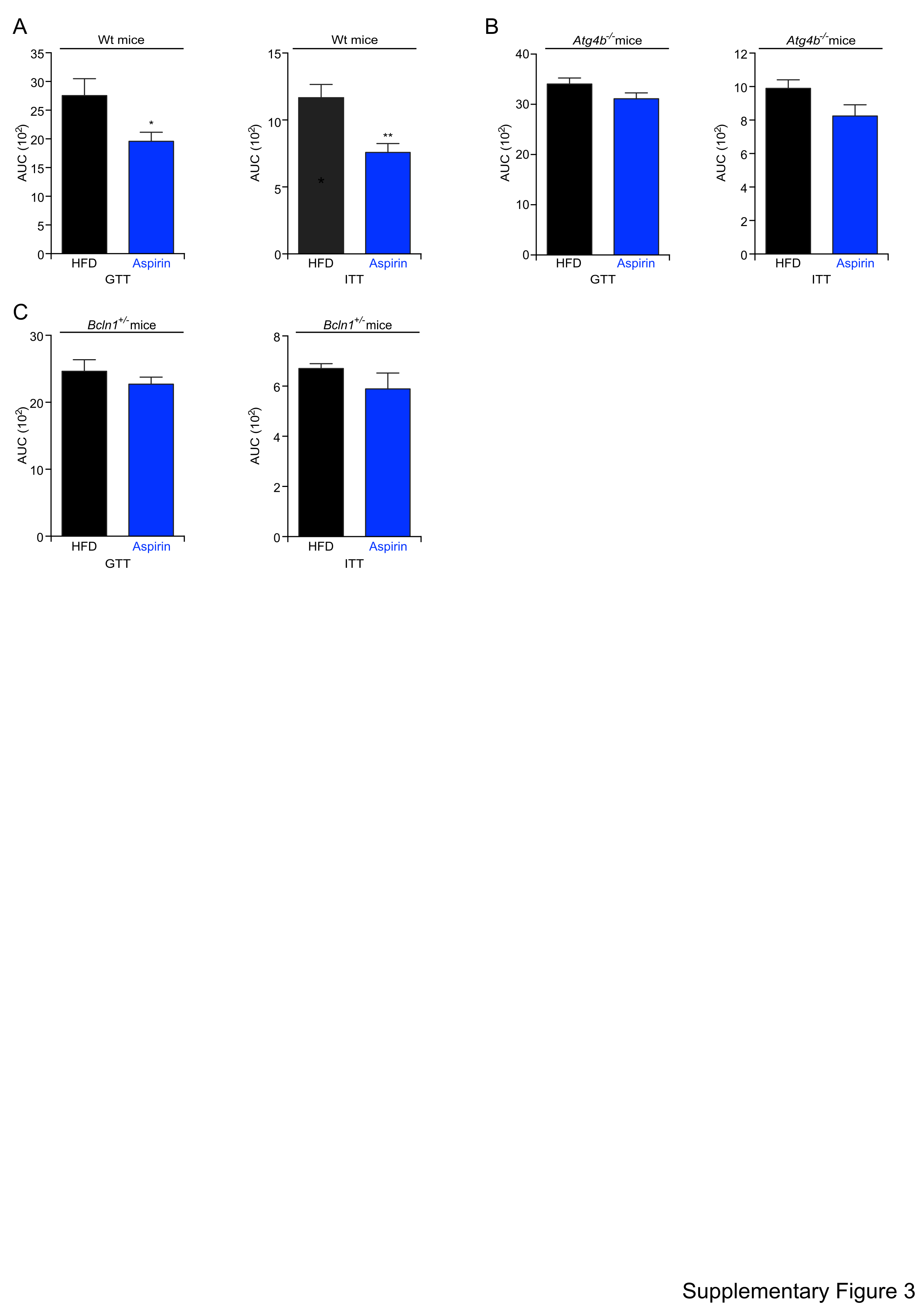

Supplement: Supplementary file 4 — Figure S3 [file 41420_2020_365_MOESM4_ESM.png]
