# Supplementary material for: Autophagy-mediated metabolic effects of aspirin
Source: Cell Death Discov. 2020 Nov 24;6:129. doi: 10.1038/s41420-020-00365-0 (PMC7687910; doi:10.1038/s41420-020-00365-0)
Supplement: Supplementary file 7 — Table S3 [file 41420_2020_365_MOESM7_ESM.pdf]

Data are the average of at least 3 different experiments
Statistical analysis were done by two tails paired Student *t* test

	DMEM-Ctrl	Salic 5mM
UNR	0,997947071	1,121132286
aENOLASE	1,434304667	1,542104
ANP32B	0,911579567	1,0567602
ARFGAP	0,998200533	1,117442667
BAG6	0,854731667	0,961118
CASP8AP	1,234189	1,27614625
EP400	0,936893525	0,989320275
HISTH2B	1,021528375	1,10694015
HMBS	1,0882584	1,147064
HMGA1	1,048619933	1,144837933
HNRNPU	0,990596133	0,9796845
HSP90AB1	1,040502	0,991498667
KRT8	1,035183	1,161479667
Lasp1	0,9193596	1,001853267
SSB	1,284672667	1,314568667
MALM1	1,0759415	1,18784275
P4HB	0,9060229	1,0485325
PDIA4	1,0248694	1,155662
PTMS	0,924508233	1,0166021
SKIV2L2	0,9961571	1,1140877
STARD9	0,966870933	1,150955967
TCOF1	0,7210078	0,79847545
USP7	1,022848433	1,223028
ZNF302	1,0350381	1,15124825

p value Ctrl siRNA *vs* Ctrl UNR		*p* value Salicylate 5mM *vs* Ctrl siRNA
		0,000000002655
0,112333078		0,30857794
0,212015596		0,029363159
0,978828835		0,045903441
0,097228059		0,320144139
0,063663076		0,298800704
0,498028353		0,253553225
0,637069857		0,171440197
0,178923363		0,032247851
0,490296026		0,384982439
0,836199553		0,42999421
0,288489212		0,393519763
0,583506244		0,057790258
0,299215925		0,013110914
0,017447695	#	0,52084851
0,107201536		0,033296012
0,395538142		0,104615827
0,567439927		0,008644972
0,36443491		0,016776137
0,948944903		0,114547884
0,584708621		0,191864466
0,026299837	#	0,135850336
0,855128648		0,168619401
0,137653359		0,154272773
